# Delayed Neurosurgical Intervention in Traumatic Brain Injury Patients Referred From Primary Hospitals Is Not Associated With an Unfavorable Outcome

**DOI:** 10.3389/fneur.2020.610192

**Published:** 2021-01-13

**Authors:** Niklas Grevfors, Caroline Lindblad, David W. Nelson, Mikael Svensson, Eric Peter Thelin, Rebecka Rubenson Wahlin

**Affiliations:** ^1^Division of Perioperative Medicine and Intensive Care (PMI), Department of Anesthesiology, Karolinska University Hospital, Stockholm, Sweden; ^2^Department of Clinical Neuroscience, Karolinska Institutet, Stockholm, Sweden; ^3^Section of Anesthesiology and Intensive Care, Department of Physiology and Pharmacology, Karolinska Institutet, Stockholm, Sweden; ^4^Department of Neurosurgery, Karolinska University Hospital, Stockholm, Sweden; ^5^Department of Neurology, Karolinska University Hospital, Stockholm, Sweden; ^6^Department of Clinical Science and Education, Södersjukhuset, Karolinska Institutet, Stockholm, Sweden; ^7^Department of Anesthesia and Intensive Care, Södersjukhuset, Stockholm, Sweden; ^8^Ambulance Medical Service in Stockholm (Ambulanssjukvården i Storstockholm AB), Stockholm, Sweden; ^9^Academic EMS, Stockholm, Sweden

**Keywords:** traumatic brain injury, secondary referral hospital, pre-hospital management, human, neurosurgery

## Abstract

**Background:** Secondary transports of patients suffering from traumatic brain injury (TBI) may result in a delayed management and neurosurgical intervention, which is potentially detrimental. The aim of this study was to study the effect of triaging and delayed transfers on outcome, specifically studying time to diagnostics and neurosurgical management.

**Methods:** This was a retrospective observational cohort study of TBI patients in need of neurosurgical care, 15 years and older, in the Stockholm Region, Sweden, from 2008 throughout 2014. Data were collected from pre-hospital and in-hospital charts. Known TBI outcome predictors, including the protein biomarker of brain injury S100B, were used to assess injury severity. Characteristics and outcomes of direct trauma center (TC) and those of secondary transfers were evaluated and compared. Functional outcome, using the Glasgow Outcome Scale, was assessed in survivors at 6–12 months after trauma. Regression models, including propensity score balanced models, were used for endpoint assessment.

**Results:** A total of *n* = 457 TBI patients were included; *n* = 320 (70%) patients were direct TC transfers, whereas *n* = 137 (30%) were secondary referrals. In all, *n* = 295 required neurosurgery for the first 24 h after trauma (about 75% of each subgroup). Direct TC transfers were more severely injured (median Glasgow Coma Scale 8 vs. 13) and more often suffered a high energy trauma (31 vs. 2.9%) than secondary referrals. Admission S100B was higher in the TC transfer group, though S100B levels 12–36 h after trauma were similar between cohorts. Direct or indirect TC transfer could be predicted using propensity scoring. The secondary referrals had a shorter distance to the primary hospital, but had later radiology and surgery than the TC group (all *p* < 0.001). In adjusted multivariable analyses with and without propensity matching, direct or secondary transfers were not found to be significantly related to outcome. Time from trauma to surgery did not affect outcome.

**Conclusions:** TBI patients secondary transported to a TC had surgical intervention performed hours later, though this did not affect outcome, presumably demonstrating that accurate pre-hospital triaging was performed. This indicates that for selected patients, a wait-and-see approach with delayed neurosurgical intervention is not necessarily detrimental, but warrants further research.

## Introduction

Traumatic brain injury (TBI) is a considerable public health problem globally, and approximately 5.5 million people suffer a severe TBI annually (1). The estimated incidence in Europe is 262/100,000 of patients admitted to hospital, with an average-related mortality of 11/100,000 (2, 3) and many survivors living with life-long disabilities, resulting in substantial costs for society (4).

Actions taken in the pre-hospital setting focus primarily on preventing secondary insults, such as hypotension, hypothermia, and hypoxia, which have been shown to be detrimental to TBI patients, as they may aggravate secondary brain injury development (5–7). While it is possible to initiate resuscitation at the scene of accident (SoA), it is of key importance to reach a hospital for diagnosis and intervention (8). Furthermore, prolonged pre-hospital time, as well as long distances to the hospital, has been shown to be associated with unfavorable outcome (9–11). Transport destination is often based on local guidelines, where unconscious patients with deranged physiological parameters generally will be transferred directly to level 1 trauma centers (TCs), whereas patients with less severe injuries are taken to tertiary hospitals for diagnostic work-up (12, 13).

Many patients suffering from milder TBI will not require neurosurgical interventions and/or monitoring in neurosurgical departments (14) and, thus, do not necessarily need direct referral to a level 1 TC. However, in severe TBI patients (unconscious at the SoA), increased intracranial pressure (ICP) is present in up to 70% of patients (15), a central metric in the management of moderate-to-severe TBI patients requiring either monitoring or evacuation surgery (16). Studies have shown that TBI patients with delayed surgery have worse outcome as compared with those with early interventions (17, 18). Further, in patients where decompressive hemicraniectomy is warranted, the time to surgery might be of importance (19). Compared with patients who have rapid surgery, an un-operated patient could have an ongoing intracranial mass-effect and, thus, a longer burden of increased ICP and lower cerebral perfusion pressure (CPP), which have been shown to affect outcome (20). Thus, affected patients suffering from mass occupying traumatic lesions should benefit from transportation directly to a designated TC where rapid diagnostics and potential neurosurgical intervention may take place (21, 22).

In the literature, studies comparing direct vs. secondary transfers of TBI patients to appropriate TCs in large conclude that for secondary TC referrals, there is a delay in transfer times and longer dwell times in the emergency rooms (ERs) before patients are adequately managed (21–23), with some studies demonstrating that these delays result in worse outcome (24, 25). However, a systematic review showed no difference in mortality between patients who were directly transferred to TCs or to non-TCs (26). Furthermore, sending too many patients with suspected TBIs directly to TCs resulted in substantial over-triaging (27), with erroneous allocations of resources. This stresses the need for primary hospitals to perform diagnostic work-ups on the majority of TBI patients who are not in the severe category and might require neurosurgical care. However, in major trauma, not specifically TBI, potentially dangerous under-triaging occurs in about 11–22% (28, 29) of cases; thus, numerous patients are at risk of not getting the level of care they require expeditiously. While different delays have been shown between direct and secondary transfers, we have not been able to identify any studies that look specifically at delayed neurosurgical intervention (time from trauma to surgery) and its effect on functional outcome in the emergency setting.

Therefore, this study aims to primarily investigate the long-term outcomes between TBI patients who were primarily or secondarily transported to a neurosurgical unit, specifically looking at the time from trauma-to-surgery in the two groups.

## Materials and Methods

### Ethics

The study received ethical approval from the Regional Ethical Review Board in Stockholm with reference numbers 2007/1113-31 (with follow-up amendments 2010/1979-32, 2013/1718-32, and 2014/691-32), as well as 2015/1675-31/1. The ethical review board waived the need for informed consent.

### Study Design and Population

This was a retrospective observational cohort study. Inclusion criteria were: adults and late adolescent trauma patients (>14 years old) with existing pre-hospital charts, documented TBI on a head/brain computed tomography (CT) scan [International Classification of Disease (ICD)-10 S06.2–S06.9], and treated at the Department of Neurosurgery at the Karolinska University Hospital (KUH). Exclusion criteria were: patients admitted to the reporting hospital [TC or non-trauma center (NTC)] >6 h after the trauma, cases where the reported time of the trauma was unclear or unknown, patients transferred to the KUH >24 h after admission to any other hospitals, or patients transported from another county to the KUH.

Patients were included during the period of 1st of January 2008 to the 31st of December 2014 in Region Stockholm, Sweden. This data set has previously been used to study the clinical efficacy of pre-hospital intubation (30).

### Trauma Organization and Pre-hospital Data Collection

The structure of hospital trauma care in Scandinavia is similar to the American College of Surgeons trauma level system (31), a classification system for hospitals receiving trauma patients. This is based on the level of care that the hospital can provide where, e.g., a level 1 (highest care) hospital can provide neurosurgical and neurointensive care at all times. Region Stockholm consists of seven emergency departments and has a population of about 2.3 million. KUH is the only hospital in the region serving functionally as a level 1 trauma hospital.

In 2008, new pre-hospital guidelines were implemented regionally in order to better accurately triage of more severely injured patients directly to the TC (13, 32). The algorithms therein base TBI triage on known TBI outcome predictors that have been identified primarily on direct TC cohorts, including low Glasgow Coma Scale (GCS) (3–13, severe-to-moderate TBI), unresponsive pupils, hypotension (<90 mmHg), hypoxia (<90% saturation), or respiratory rate <10 or >29 per min, and penetrating injuries or presence of extracranial multi-trauma (13).

The studied region consists of approximately 6,500 km^2^, including an archipelago of over 30,000 islands. At the time of the study, the organization of the emergency medical services (EMSs) in Region Stockholm included one publicly owned company and two private contractors coordinated by one Emergency Medical Communications Center. Between 07:00 and 20:00 (i.e., daytime), there were 55–61 ground ambulance and three rapid-response vehicles operating in the area (33). The ground-based ambulance crew consisted of an emergency medical technician (EMT) and a registered nurse. One of the rapid-response vehicles was physician-manned and the two others by a nurse anesthetist and an EMT. During nights, there was no physician on duty, and there were about 38 functioning ambulances in the area. Furthermore, the region also had one helicopter (and one additional helicopter during summer) manned by a nurse anesthetist and one mobile intensive care unit (ICU) for transfers between critical care units.

### Clinical Variables

Data were extracted from the neuro-trauma registry at KUH. The pre-hospital network (CAK-net) used by all pre-hospital staff during the study period was used to extract the pre-hospital data. The ambulances are rigged with a global positioning satellite system (GPS) that supplies a GPS coordinate according to the SWEREF 99 (Swedish reference frame 1999) system (34).

Gender and age were extracted from hospital charts. Time from arrival at the scene to hospital arrival was extracted from the pre-hospital charts, as well as systolic blood pressure (SBP), respiratory rate, and GCS on the scene and during transport. The distances and time periods were defined as follows: distance from the SoA to hospital was defined in kilometers, and the time on the scene and the departure from the scene until hospital arrival were defined in minutes and seconds. The presence of multi-trauma was noted, defined as significant injury to any other major organ systems except the spine and head as per previous definitions (35). If available, it was noted if it was a high energy trauma as defined by the Advance Trauma and Life Support (ATLS) guidelines (36). At the SoA, GCS was recorded, and if not specified, “unconscious” patients were defined as GCS 3–8 (37). “Pupil unresponsiveness” was categorized as one or two pupils presenting without a light reflex. To evaluate the neuro-radiological damage, we assessed the primary CT scans according to Rotterdam CT scores (38), Marshall classification (39), and Stockholm CT scores (40). The Stockholm CT scores have been shown to exhibit best correlation to outcome; therefore, we used it in our analysis (41). Furthermore, head abbreviated injury score (AIS) [version 2005 update 2008 (42)] was noted, together with injury severity score (ISS) and new injury severity score (NISS). S100B, a protein biomarker of brain tissue fate (43), was sampled at admission and every 12 h at KUH. Samples from admission and after 12 h following TBI (as these samples have been shown to be less affected by external trauma) were registered (44, 45). Survival status and 30-day mortality were noted, as well as the length of stay in the critical care unit. Surgical intervention was included and defined as the primary surgical intervention ICD code. Long-term outcome was assessed by clinic visits and questionnaires concerning health-related quality of life and was used to extract functional outcome at 6–12 months using the five stages of the Glasgow Outcome Score (GOS) (37).

### Statistical Analysis

In describing patient demographics, categorical data are presented as count (percentage) and continuous data as medians (interquartile range). Group-wise comparisons were conducted using χ^2^/Fisher's exact test and Mann–Whitney *U*-test for categorical and continuous variables, respectively. For outcome predictions, multivariable logistic or else proportional odds regression was used with either dichotomized or the full-scale GOS (proportional odds) (using the “rms” package in R) (46) as a dependent variable. The proportional odds analyses are available as Data Sheet 1. The International Mission for Prognosis and Analysis of Clinical Trials in TBI (IMPACT) study group has previously shown that the proportional odds analyses are adequate to use in TBI cohorts (47), so even if the proportional odds assumption does not necessarily apply in our cohort, it could be considered an analysis of a larger group where these assumptions are true.

An aim of the study is to focus on signs and symptoms available first during the initial triage at the SoA in a cohort that is later deemed in need of neurosurgical treatment or surveillance and evaluate the initial decision-making and triage in relation to choice of hospital and later outcomes, also comparing this with later available information at admission. Inherently, the patient cohort immediately transferred to the TC was likely to differ from patients secondarily transferred. We approached this expected confounding *via* two different analytical approaches. Firstly, we performed multivariable analysis adjusting for known predictors and potential confounders using variables in the IMPACT TBI model by Steyerberg et al. (48). This approach uses late available information, such as data from CT scans, to investigate if “secondary referral” was a significant predictor of outcome. Secondly, we employed propensity score estimation modeling where “secondary referral” was used as dependent variable in a logistic regression model. Here, we did *not* include metrics that could not be assessed at the SoA (such as radiological variables) or variables relating to hospital proximity, as those do not form part of the guidelines (48). This approach will inherently focus the analysis in the matched sample on an intermediate group that has similar presentation at the SoA but is triaged differently. For this, we utilized the pre-hospital guidelines available but extended our model somewhat (13). Independent variables comprised all pre-hospital vital parameters, neurological features that can be determined and were recorded at the SoA, and trauma characteristics. In the pre-hospital setting, GCS motor score at the SoA was frequently missing. Whenever we had a SoA GCS of four, we assumed the motor score to be two, representing the “best possible” motor score. Equivalently, when SoA GCS was 14, we assumed that GCS motor score was six. There was also limited documentation of pupillary reactions, prior to the arrival at the TC. For the final propensity score estimation, variables included were: respiratory rate, pulse, oxygen saturation, GCS, multi-trauma, low-energy trauma [according to the Utstein definition (49)], compromised airway, and age (where all variables except accurate age were possible to determine at the SoA, though the EMS would know if the patient was young or old). Using propensity scores, we created a balanced patient subset (*n* = 128 out of 275) using the MatchIt package in R (50), using a nearest neighbor matching algorithm. Following this, group balance was still not perfect when we added a maximum propensity score distance on the observations (51). Thereby, we obtained balanced groups, as deemed through inferential testing and graphical observation. The propensity scores are available as Data Sheet 1.

Due to missing data in several pre-hospital variables, we employed multiple imputation (*n* = 7) of our data using the “mice” package in R (52). For each imputation, we recalculated the inferential operations stated above, before pooling results estimate. Our propensity score estimation approach on the imputed data has been deemed superior in the context of inverse probability of treatment weighting as compared with other methods (53, 54). We employed this approach as it adhered with the idea of multiple imputation, but some caution is warranted when interpreting these results as it risks interfering bias. Finally, for each imputation, outcome analysis was calculated using proportional odds regression. For these analyses, we employed the MASS package in R (55), as it allows for pooling regression outputs of all imputed data. All statistical calculations were performed in the program R with the interface program R-studio (56).

## Results

### Included Patients

**Figure 1:**
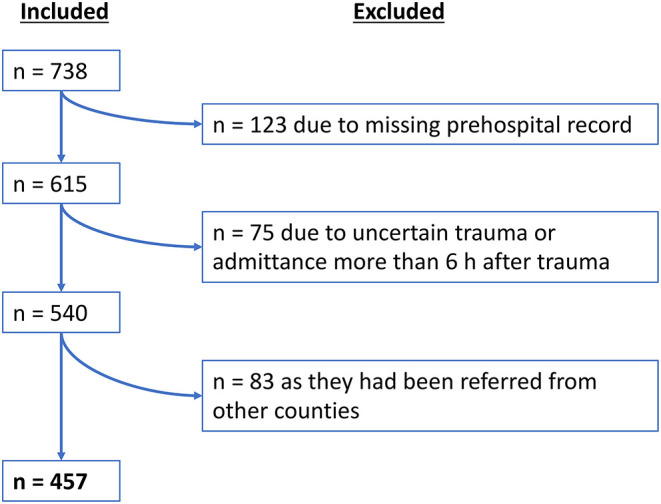
Inclusion flowchart.

During the inclusion period, a total number of *n* = 738 TBI patients were admitted to the KUH in need for neurosurgical management. A total of *n* = 457 patients met the inclusion criteria (see flowchart in Figure 1). Of those 457 patients, *n* = 320 (70%) patients were directly transported to the TC, whereas *n* = 137 (30%) patients were initially transported to another primary hospital before undergoing a secondary transfer to the TC.

### Comparison Between Direct and Secondary Transfers

The descriptive analysis of the included patients is described in Table 1. The pre-hospital data suggest a tendency toward older patients more frequently being transferred to the non-TCs and the TC receiving younger, more unstable, and more highly injured patients (e.g., higher frequency of pre-hospital hypotension, unconsciousness, high respiratory rate, high trauma energy, multi-trauma, and low GCS). Hospital data showed that patients transported to the TC had a higher incidence of unresponsive pupils and higher ISS/NISS. While S100B levels at admission were higher in the TC, Stockholm CT score and peak levels of S100B sampled after the initial phase were similar between the two cohorts.

**Table 1:** Patient demographics.

**Variable**	**Direct transfer to TC**		**Secondary transfer**		***p*-value**	**Adjusted *p*-value**
	***N*** **=** **320**		***N*** **=** **137**			
	**Data**	**Missing**	**Data**	**Missing**		
**Patient demographics**						
**Demographics**						
Age (years)	47 (26–63)	0 (0)	56 (39–64)	0 (0)	0.010	0.20
Male	231 (72)	0 (0)	103 (75)	0 (0)	0.57	1
**Pre-hospital data**						
GCS SoA	8 (4–13)	0 (0)	13 (11–14)	3 (2.2)	<0.001	**<0.001**
Unconscious SoA	164 (51)	0 (0)	14 (10)	0 (0)	<0.001	**<0.001**
Hypotension SoA	10 (3.1)	51 (16)	0 (0)	45 (33)	0.070	1
Hypoxia SoA	34 (11)	43 (13)	3 (2.2)	47 (34)	0.014	0.28
High-energy trauma	98 (31)	144 (45)	4 (2.9)	106 (77)	<0.001	**<0.001**
Multi-trauma	117 (37)	0 (0)	14 (10)	0 (0)	<0.001	**<0.001**
**Hospital data**						
Pupil unresponsiveness	63 (20)	6 (1.9)	15 (11)	3 (2.2)	0.03	0.58
Stockholm CT score	2.1 (1.5–3.1)	0 (0)	2 (1–3)	1 (0.73)	0.015	0.30
S100B peak 12–36 h (μg/L)	0.3 (0.19–0.59)	69 (22)	0.2 (0.1–0.57)	52 (38)	0.017	0.35
S100B admission (μg/L)	2.1 (0.87–5.0)	136 (43)	0.34 (0.21–1.45)	114 (83)	<0.001	**<0.001**
AIS > 3	249 (78)	3 (0.93)	99 (72)	4 (2.9)	0.38	1
ISS	25 (17–30)	3 (0.93)	19 (16–25)	4 (2.9)	<0.001	**<0.001**
NISS	41 (27–50)	3 (0.93)	29 (24–38)	4 (2.9)	<0.001	**<0.001**
Surgery	251 (78)	0 (0)	103 (75)	0 (0)	0.46	1
**Outcome data**						
In-hospital mortality	37 (12)	0 (0)	13 (9.5)	0 (0)	0.62	1
NCCU LoS	6.0 (1.1–16)	0 (0)	1.4 (0–6.7)	0 (0)	<0.001	**<0.001**
TC LoS	16 (7–30)	0 (0)	8 (4–16)	0 (0)	<0.001	**<0.001**
Unfavorable GOS	131 (41)	0 (0)	46 (34)	0 (0)	0.14	1

*Data are presented as median (IQR) or count (percentage) for continuous and categorical data, respectively. p-values were calculated using either Fisher's exact test (categorical data) or Mann–Whitney U-test (continuous data). Bonferroni correction was applied for multiple testing and is presented as adjusted p-values. Significant adjusted p-values in bold*.

*AIS, abbreviated injury score; CT, computed tomography; GCS, Glasgow Coma Scale; GOS, Glasgow Outcome Scale; IQR, interquartile range; ISS, injury severity score; LoS, length of stay; NCCU, neuro-critical care unit; NISS, new injury severity score; SoA, site of accident; TC, trauma center*.

There was no difference in the hospital length of stay, whereas the ICU length of stay was longer in the group initially transported to the TC. Approximately 75% of the patients in both direct referrals and secondary transports had surgery performed. The primary surgical intervention differed somewhat between the two groups (Table 2), but neurosurgical hematoma evacuation surgeries and monitoring surgery dominated in both. The incidence of evacuated acute subdural and epidural hematomas (EDHs) was higher in the secondary transfer group. Insertion of intracranial monitoring surgery (ICD AAA99) was the primary initial surgery (Supplementary Figure 1), though often in conjecture with acute subdural hematoma (SDH) evacuation, but generating two separate sub-ICD codes. No unadjusted differences between the groups were seen in-hospital mortality or long-term GOS (Table 1).

**Table 2:** Type of surgery performed.

**Type of surgery**	**Direct to TC, 251 procedures**	**Secondary referrals, 103 procedures**
**Type of primary surgical intervention performed, divided by transportation type**		
Evacuation of acute subdural hematoma	71 (28%)	47 (46%)
Ventriculostomy (external ventricular drain)	51 (20%)	8 (8%)
Placement of intracranial pressure device	46 (18%)	3 (3%)
Evacuation of epidural hematoma	31 (12%)	23 (22%)
Evacuation of traumatic intracerebral hematoma (contusions)	7 (3%)	6 (6%)
Revision of skull fracture	6 (2%)	5 (5%)
Revision of penetrating or perforating head injury	5 (2%)	4 (4%)
Microsurgical discectomy of the cervical spine	3 (1%)	0
Other neurosurgical interventions	14 (6%)	4 (4%)
Other non-neurosurgical interventions	17 (7%)	3 (3%)

### Functional Outcome Between Transfer Groups

Multivariable logistic regression showed no significant difference in dichotomized long-term functional outcome (GOS) between the group that was transported directly to the TC compared with the group that was secondarily transported to the TC (*p* = 0.140 unadjusted, *p* = 0.297 adjusted for age, pupil responsiveness, admission GCS, and Stockholm CT score). Similarly, a proportional odds analysis, using all levels of GOS, showed a non-significant trend toward more favorable outcome in patients who were secondarily transferred (*p* = 0.062 unadjusted, *p* = 0.32 adjusted for the same parameters above) (Supplementary Data 1).

### Transportation and GCS Dynamics

Patients transported directly to the TC had a similar total pre-hospital time (time from reported trauma to arrival at the hospital) as compared with patients not transported directly to the TC, 45 vs. 44 min, respectively. However, the on-scene time (29 vs. 26 min) and the distance (at average almost 4 km longer) were longer for the direct-to-TC transfers (Table 3). The time from trauma to initial CT scan (performed either at the TC or at the primary hospital) was at average approximately an hour longer for patients with non-direct TC transfers (Table 3).

**Table 3:** Pre-hospital transportation distance and times.

**Variable**	**Direct transfer to TC** ***N*** **=** **320**		**Secondary transfer** ***N*** **=** **137**		***p*-value**	**Adjusted *p*-value**
	**Data**	**Missing**	**Data**	**Missing**		
**Pre-hospital and hospital** **Δtimes and** **Δdistances**						
Distance SoA to PH (km)	13 (6.5–25)	0 (0)	9.1 (3.5–15)	0 (0)	<0.001	<0.001
Time at SoA (min)	29 (23–42)	4 (1.3)	26 (20–37)	1 (0.73)	0.012	0.14
Time from trauma to arrival PH (min)	45 (35–61)	1 (0.31)	44 (36–60)	10 (7.3)	0.77	1
Time from trauma to CT (h, min)	1 h 22 min (1 8–1 h 43 min)	1 (0.31)	2 h 29 min (1 36–4 h 5 min)	1 (0.73)	<0.001	<0.001
Time to surgery (h, min)	3 h 39 min (2 31–7 h 30 min)	72 (23) (no surgeries performed)	8 h 47 min (5 25–16 h 10 min)	36 (26) (no surgeries performed)	<0.001	<0.001

*Time and distance calculations are presented as median (interquartile range), unless otherwise stated. p-values were computed using the Mann–Whitney U-test, and subsequent Bonferroni correction was applied (yielding adjusted p-values)*.

*CT, computed tomography; PH, primary hospital; SoA, site of accident*.

GCS was already low in a majority of patients directly transferred to the TC (52%) (Table 4). The share of patients who were unconscious increased dramatically in the non-direct TC transfers from the primary hospital to admission at the TC (11–33%) (Table 4). While some represent “true” deteriorations, some may include intubations (or probably both).

**Table 4:** Dynamics of Glasgow Coma Scale over time.

	**Scene of accident**	**Primary hospital**	**Admission trauma center**
**Secondary referrals**			
Median GCS (IQR)	13 (12–14)	13 (12–14)	13 (4–14)
Percentage GCS 3–8	12%	11%	33%
Missing (*n*, %)	14 (10%)	9 (7%)	6 (4%)
**Direct referrals**			
Median GCS (IQR)	8 (4–13)	NA	7 (3–13)
Percentage GCS 3–8	52%	NA	54%
Missing (*n*, %)	20 (6%)	NA	12 (4%)

*GCS dynamics from scene of accident, primary hospital (for secondary referrals), and at admission to trauma center*.

*GCS, Glasgow Coma Scale; IQR, interquartile range*.

### Outcome Related to Delayed Surgeries

**Figure 2:**
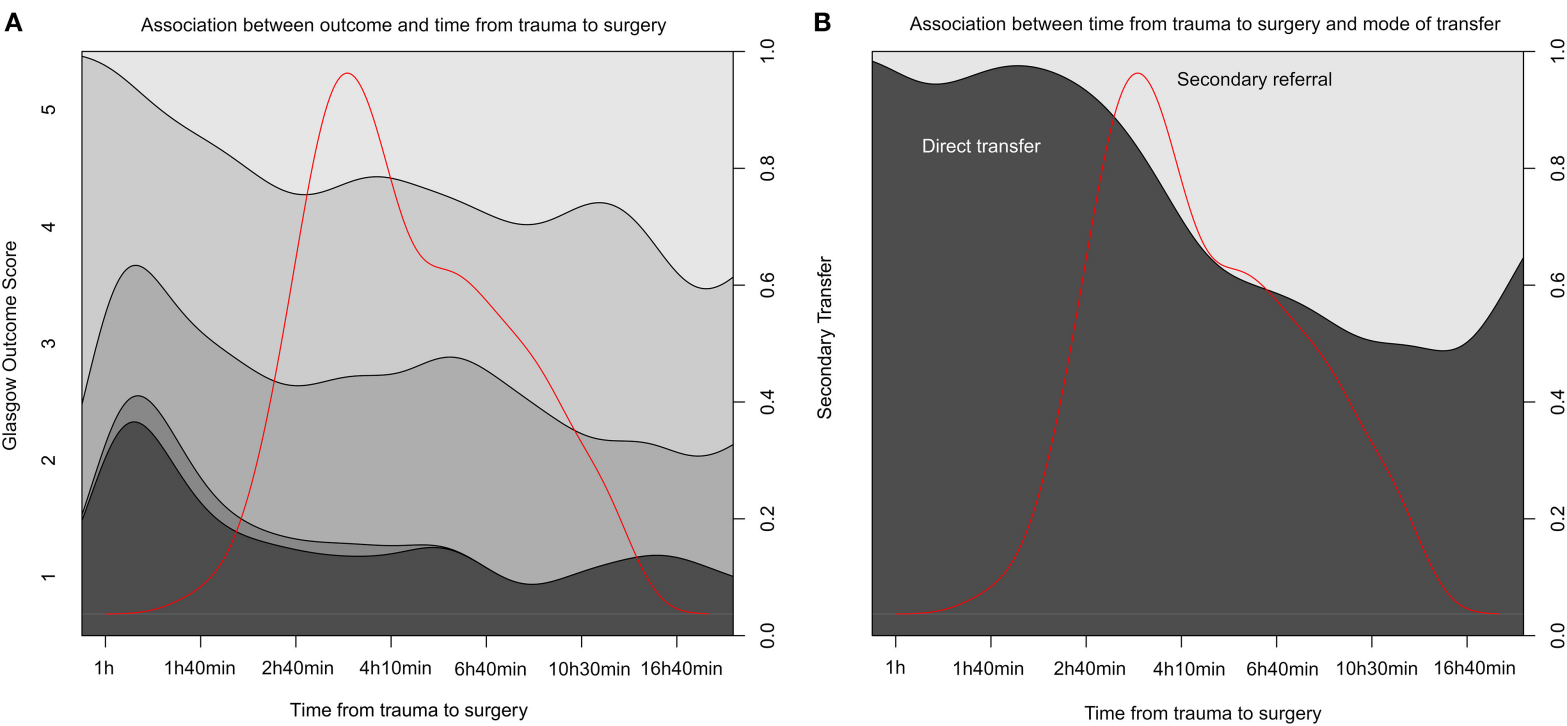
Time from trauma to surgery and association to outcome and transportation mode. **(A)** A conditional density (CD) plot of the different stages of Glasgow Outcome Score (y-axis left) over time between trauma and surgery (x-axis) for the first 24 h after injury. y-Axis right summing the proportion to one. **(B)** A similar CD plot of the cohort divided by transportation mode (direct or secondary referrals) on y-axis left. The red line in both graphs illustrates data distribution. Data is logged to visualize it more clearly.

The direct transports had a median time from trauma to surgery of 3 h 39 min, whereas, patients with secondary transports commenced surgery at 8 h 47 min following the reported trauma (Table 3). A univariate analysis looking at a subgroup of patients with surgeries within the first 24 h of trauma (*n* = 295) actually suggests a significant positive correlation between better outcome and a more extended time between trauma and surgery (*p* = 0.023) (Figure 2A; Supplementary Data 1). This can be assumed due to confounding by indication. However, and more importantly, there was no increase in unfavorable outcomes as time-from-trauma to surgery progressed up until 24 h after injury. The early negative association of early surgeries toward outcome was maintained even when adjusting for the group that was secondarily transferred (*p* = 0.043). However, if adjusted for known TBI predictors (age, pupil responsiveness, admission GCS, and Stockholm CT score) and secondary transfer, time between trauma and surgery was no longer significant (*p* = 0.431). In an exploratory approach, we isolated subdural and EDH evacuation surgeries as these could be considered the most relevant to perform as early as possible after trauma, but the results remained similar (data not shown).

We executed propensity score analysis followed by multiple imputation and then re-did these analyses on imputed, matched samples. The multiple imputation data sets showed adequate distributions in comparison with available data, which were also supported by the imputation index sample and imputation quality plots (Supplementary Figures 2–4). Univariate proportional odds regression, pooled for all imputations, once again demonstrated that outcome assessed by GOS among the subset of patients operated on within 24 h did not depend on time to surgery (estimate = 0.0007, *p* = 0.204). Hence, this highlights inherent differences between the two groups that when accounted for using propensity score estimation did not translate to differences in outcome. Figure 2B shows the distribution of surgery initiation depending on if the patient was directly transferred to the TC or admitted as a secondary referral.

## Discussion

Despite differences ranging several hours between trauma and neurosurgery in patients directly transferred to TC and secondary transfers, we found that neither long-term GOS nor mortality was significantly different between the two groups. These results remained, before and after adjusting for known severity predictors, or propensity matching cohorts. Late deterioration and surgery appear, thus, to trigger adequate responses in this setting, and the longer time from trauma to surgery in the transfer group does not appear crucial in this study. A possible relevant metric to explore in future studies would be time from clinical deterioration to surgery, a potential outcome metric that could be affected by transfer times. Interestingly, the transferred group is a selected group that deteriorates as to require transfer and would be expected to do worse than variable adjustment from the initial (prior deteriorating) variables would predict. This does, however, not seem to be the case, again suggesting that late deterioration may be a slower process that can be handled adequately. In aggregate, we cannot identify secondary transports or delayed surgery to be associated with increased risk. However, this observational retrospective data set requires specific discussion.

### Triage

Triage at the SoA will be responsible for major differences in the TC and transfer cohort. The patients who were directly transferred to the TC were younger, more often unconscious, hypotensive, and had abnormal pupillary responses. That these TBI patients are prioritized for direct transfers are in line with several other studies (21–23, 57) and follow established triaging guidelines implemented in the region (13). This is supported by an earlier analysis of this cohort (30), where triage concerning on site intubation closely followed local guidelines. Similarly, propensity scoring indicated that pre-hospital clinical variables were different between patients at the SoA. Our pre-hospital trauma guidelines stipulate that blood pressure, respiratory rate, mechanism of injury, and GCS should direct pre-hospital triaging (13, 32). In addition, we found that low-energy trauma, multi-trauma, and heart rate were important parameters. Even though this study does not allow for causation, it might be interpreted as an indication that additional factors may be usable in the pre-hospital setting. Aside from this, the triaging was performed in accordance with established guidelines, so while the amount of secondary transports was high, it is difficult to say if it is due to actual under-triaging or not. It should be acknowledged that the pre-hospital care of TBI in Europe is very heterogeneous, as is highlighted by data and results from the CENTER-TBI consortium (58, 59). Novel markers that could improve accurate triage are, thus, needed and could help design better triaging protocols in the future.

Despite generally correct triage, the AIS, as well as Stockholm CT score, did not differ between the two groups. As Head-AIS is assessed during the treatment period (and Stockholm CT score following the admission CT scan), and not on site, this is in line with that the arriving EMS observes and acts on clinical signs and measurements at the SoA that are incomplete as to distinguish TBI severity. This is also reflected in the propensity matching. Furthermore, both the AIS-derived indices NISS and ISS were higher in the direct transfer cohort, likely due to the higher degree of multi-trauma in the TC group. The S100B levels reflected an interesting pattern with early levels being markedly higher in the direct TC group. While this is presumably due to a higher injury burden from both intracranial and extracranial sources (44, 60), the group with secondary referrals had their samples acquired on average hours later, which also has been shown to result in lower levels due to rapid serum clearance in the absence of ongoing injury (61, 62). However, 12–36 h samples, better corresponding to the brain injury (44), were relatively similar between the two groups, suggesting again that the cerebral injury burden was perhaps more similar for the patients in need of neurosurgical management for the first 24 h after injury, but not identifiable during early triage. Additionally, GCS decreased among some patients in the secondary referral cohort while at the primary hospital. It is possible that sedation was initiated, and that intubation was performed, in order to prepare for surgery, hence resulting in a GCS of three when arriving at the TC.

Our interpretation is that while the group directly transferred to the TC had more severe symptoms at the SoA, the fact that AIS, Stockholm CT score, and S100B were not significantly different between the groups shows that the radiological and biochemical aspects of the injury were similar when the admission CT was performed. Another possibility is that the older cohort that later needed secondary transfer to the TC had similar injuries in terms of mass lesions size and severity, but they were less affected neurologically by them at the SoA. Though, the delay at the primary hospital from trauma to CT scan could also play a role as lesions may have significantly progressed.

This study illustrates a good example of using a CT scoring system and brain tissue damage markers to establish a more objective baseline stratification between two cohorts, showing that while different in symptomatology, the cohorts shared similarities related to their intracranial pathology. It also demonstrates that serial measurements of S100B and other markers could be used to monitor cerebral deterioration (62), although currently, this is only performed at the TC in our region.

In summary, direct transfers were performed on patients who were deemed to have more severe injuries at the SoA, but patients in need of neurosurgical intervention deteriorated and had similar intracerebral injuries during the first 24 h. In aggregate, the cohorts are intrinsically different as expected by triage, and as such, multivariable adjustment and propensity matching are required to match and compare groups.

### Secondary Transfers

Secondary transfers appear relevant as nearly 75% of the patients, in both groups, eventually needed surgical interventions of some kind. While ultimately suffering from brain hemorrhages requiring surgery, many of the secondary referrals did not present with signs of severe injuries at the SoA and, thus, had progressive injuries that were later detected at the non-TCs. An appealing hypothesis is that among a small subset of low-energy trauma cases, injury progression occurs slowly throughout the course of stay at the non-TC. This emphasizes the need to closely monitor initially conservatively treated TBIs, as many will be eligible for delayed surgery.

We could not see that secondary transfers had worse outcome, contrasting some of the literature. In a systematic review of pre-hospital time's influence on outcome in trauma patients by Harmsen et al. (10), two studies from Dinh et al. (63) and Tien et al. (64) are highlighted. The former notice a survival effect in patients arriving >1 h 30 min, whereas the latter see that patients referred within 1 h (previously referred to as the “golden hour”) have a better outcome than patients arriving later. An additional study by Hartl et al. showed that severe (GCS 3–8) TBI patients, not transferred to a TC, had 50% higher mortality than direct TC transfers (24). Prabhakaran et al. noted similar dangers with delayed transfers, highlighting risks with prolonged pre-hospital times and subsequent dwell times in non-TC emergency departments (25).

However, similar to the systematic review on pre-hospital strategies in TBI by Pickering et al. outcome in our study was not affected if patients were transported direct to a TC or not (26), which we believe could be due to a number of factors. First, the time duration from reported trauma to hospital arrival was relatively short for both the direct and secondary referrals (45 and 44 min, respectively), with no patients even in the interquartile range falling outside 1 h. In previous studies from this cohort and northern Sweden, secondary insults during these short periods do not seem to affect outcome to the degree earlier thought (30, 65). Swedish pre-hospital data are also unique in that we report time from the reported trauma, whereas other studies most commonly report time from when EMS arrives at the scene to hospital arrival, underestimating the time since the trauma occurred. Second, almost exclusively, all “severe” traumas were directly transferred to the TC (looking at trauma energy level, presence of multi-trauma, and GCS); thus in comparison with Hartl and Prabhakaran (24, 25), we had very few patients who were severely injured that were not directly transferred. In the review by Harmsen, it is also difficult to establish exactly what non-TCs could and could not provide for the patients. While referring emergency hospitals in the Stockholm region do not have neurosurgery departments, all have a general surgeon and an anesthesiologist present in the ER when the ambulance arrives. Thus, immediate resuscitation measures may be undertaken and intubation performed, if necessary, prior to the CT scan. The study by Prabhakaran et al. also indicated that prolonged time on scene seems to be associated with an unfavorable outcome (25). In contrast, in a study by Kim et al. studying general trauma (a majority were TBI patients) from South Korea, they found that longer on-scene time significantly decreased mortality (66). This highlights that it is difficult in retrospect to analyze this, as the EMS on scene will do what is necessary in order to stabilize the patient for transportation in varying situations and settings. Age is another important aspect of this study. In TBI, older age is a key independent predictor of poor functional outcome (67, 68). The secondary transferred patients in this study showed a higher median age of 56 (vs. 47 in the TC cohort). This might contribute to the non-significant difference of poor outcome in our material, as we are comparing more severely injured, younger multi-trauma patients with older, isolated brain injury patients. However, we adjusted for this in our propensity score estimation model, why we do not believe this to cause any major residual confounding.

In aggregate, the analyses showed no outcome difference between the TC and non-TC cohorts, hence indicating correct triaging but could also be due to rapid transfer times in general and escalated therapy measures even in the non-TC cohort.

### Timing of Surgery

In addition to secondary transfers, we did not see that delayed neurosurgical interventions affected functional outcome in our TBI cohort. The relevance of time from trauma to surgery is difficult to explore in a retrospective study and presumably unethical in a prospective randomized study. However, historical TBI cohorts from the 1970s and 1980s showed that mortality increases from 30–50% to 80–90% after 4 h for acute SDH and from 17 to 65% after 2 h for EDH (69, 70). Clearly, advances in trauma management have been done since, including improved pre-hospital resuscitation, logistics, neurosurgery, and rapid diagnostics. Still, due to underdeveloped pre-hospital systems, studies from Tanzania and Uganda in recent years report high mortality (around 50%) where time from trauma to surgery can take days (17, 71). This emphasizes that delayed neurosurgical intervention for patients in need of rapid surgery is still a global problem that needs to be acknowledged. The more intricate infrastructures of modern trauma regions may make it difficult to evaluate and compare strategies. Our data seem to suggest that critical patients with more severe injuries are operated on rapidly, whereas patients less affected by their injuries are not rushed to the same extent.

That delayed surgery does not seem to increase unfavorable outcome appears in line with Joosse et al. (21). The time 2 h 30 min was also the average time from ED arrival to surgery for emergency craniectomies in a Canadian study from 2016 in patients directly transferred to the TC (72). This highlights that our median time from trauma to surgery of 3 h 39 min (ours including pre-hospital time) is within what could be considered normal for similar trauma cohorts. That time from trauma to surgery did not affect outcome is also in line with the results from Fountain et al. who noticed no difference in outcome following acute SDH surgery vs. time from trauma (>1 h 30 min or not) over a period of 20 years (73). It should also be noted that in comparison, our cohort consists not only of critical, unconscious TBI patients, in which case transportation and management is presumably performed in greater haste. In fact, the secondary transferred group had a median GCS more equivalent with mild TBI (mTBI) cohorts, and there is still scarce evidence about the long-term outcome for mTBI requiring neurosurgical interventions at a later stage. One study from Tierney et al. (18) found unfavorable neurological outcome (GOS score <4) in 44% of patients with mTBI, especially in mild TBI patients with delayed surgery. This is markedly higher than previously reported poor outcome rates (of 25%) in TBI patients (67) and is suggested to be due to anti-coagulant use and high GCS scores despite high Head-AIS/ISS in that specific study (18). In our study, the median time from trauma to surgery for the secondary transfers was more than 5 h longer (8 h 47 min), and as no difference in outcome was seen, this indicates that for selected patients, delayed surgeries with a wait-and-see approach are not necessarily detrimental. Presumably, adequate monitoring, including vigilant staff in referring hospitals providing optimized medical treatment to the patient, and an on-going consultation with the neurosurgical departments will appropriately triage correct patients for transport and surgery.

Altogether, we found that the delayed surgeries in secondary transports to the TC did not result in more unfavorable outcomes, presumably as appropriate pre-hospital management and triage systems were applied adequately, sorting correct patients to the correct level of care and identify deterioration and need of transfer in a timely manner.

### Limitations

The retrospective design is a natural limitation. Some data were missing, which were imputed in the multivariable analyses. While this could result in less robust analyses, we only draw conclusions from findings that we believe are highly significant from a statistical standpoint. Additionally, for the propensity scoring, the imputed data showed adequate distribution in the imputation index sample and imputation quality plots (Supplementary Figures 2–4), supporting the imputations. A majority of the missing data were centered around pre-hospital ambulance charts concerning blood pressure, respiratory rate, and oxygen saturation at the SoA (Data Sheet 2 and Supplementary Figure 5). It is difficult to assess if these are missing at random. Presumably, milder traumas with higher GCS are more likely to have this data missing as these are not as important to monitor, whereas in more critical patients being actively resuscitated, these will be targeted metrics and, thus, monitored more frequently.

Another key limitation by study design is that our database used for this study only includes patients who were managed by the neurosurgical department, thus patients where a neurosurgical intervention was deemed to be futile (either a too severe injury not associated with survival or an injury too mild to benefit from patient transport to the neurosurgical unit) were not included. The finding that very early surgery was associated with more unfavorable outcome (as seen in Figure 2) could be due to that some very severe cases (e.g., in young patients) were transported to the TC directly and everything was attempted, including surgery, to save them despite knowledge of a probable poor outcome. The database we used also lacks the TBI patients who never needed neurosurgical management or monitoring, i.e., with lesions that could be monitored conservatively at non-TCs. While this would constitute quite an extensive group in terms of sample size, we believe that it is more important to study patients who truly did deteriorate to the extent where neurosurgical intervention was needed.

Moreover, by definition, patients triaged to a non-TC should be less severely injured than those immediately triaged to the TC. This hampers the statistical approach, since the two groups for comparison are inherently different. In order to compensate for this, we employed propensity score modeling. This would allow us to better validate pre-hospital triaging routines, as well as our outcome analysis results. This method has been suggested to be able to compensate for approximately 90% of confounding in similar studies (74). In total, we believe this to be the most solid approach to this type of research, since it is ethically inappropriate to design a prospective study where surgery is delayed in a randomized fashion in severe TBI patients.

From the data available, we could see that while 12% reaching the non-TC primary hospital were unconscious, about 33% of this cohort was unconscious upon arrival at the TC, but more accurate details of how and when deterioration occurred were not included in the data. Deterioration of intracranial pathology has been reported to occur in up to 50% of cases (75). It is probable that both injury and clinical status deteriorated over time so that a new more accurate decision based on the current state of the patient could be made. For future studies, as previously mentioned, it would be of key interest to obtain data on all secondarily transferred TBI patients who had CT-verified intracranial injuries, in order to find if any early clinical variables are predictive of subsequent/delayed injury progression, in order to find “high-risk” patients who initially are conservatively managed. This information should include information such as comorbidities and anti-coagulants, unfortunately not available in this study, as they may play an important role in the pathophysiology of neurological deterioration (76). We hypothesize that serial protein biomarker monitoring, in addition to clinical signs and radiology, may play an important role here, as the TBI field currently is developing a panel of protein biomarkers, suitable at different stages following the trauma (62, 77). Additionally, due to the retrospective nature of the study, it is unknown if any delay was due to the patient not needing immediate surgery in contrast to if there were logistical difficulties or iatrogenic delays in patients reaching the TC in time.

We did not include the use of helicopter transport in our study, which of course would have facilitated the transportation and hence decreased the pre-hospital time. Our experience of helicopter use in this cohort is that transport will be escalated to helicopter transport if deemed necessary due to long distances (30). Further, while an ambulance might have been used for transport to the primary hospital, helicopters were sometimes used for the secondary transfers, making it difficult to study the direct benefit of them in the current study setting.

## Conclusions

Despite significant delays until neurosurgery could be initiated, this study shows no difference with regard to long-term functional outcome between TBI patients directly or secondarily transferred to the TC in a large urban area. While patients were more rapidly admitted to the nearest primary hospital, there were significant delays in trauma to CT and surgery times. The pre-hospital decision-making regarding transport destination was presumably correct in large, as more severely injured patients were transported directly to the TC.

## Data Availability Statement

The raw data supporting the conclusions of this article will be made available by the authors, without undue reservation.

## Ethics Statement

The studies involving human participants were reviewed and approved by Regional Ethical Review Board in Stockholm. Written informed consent for participation was not required for this study in accordance with the national legislation and the institutional requirements.

## Author Contributions

ET, MS, DN, and RR contributed to the conception and design of the study. ET, RR, NG, and CL organized the database. NG, DN, and CL performed the statistical analysis. NG wrote the first draft of the manuscript. CL, DN, ET, and RR wrote sections of the manuscript. All authors contributed to the manuscript revision, read, and approved the submitted version.

## Conflict of Interest

The authors declare that the research was conducted in the absence of any commercial or financial relationships that could be construed as a potential conflict of interest.
