# Supplementary figures and images for: Delayed Neurosurgical Intervention in Traumatic Brain Injury Patients Referred From Primary Hospitals Is Not Associated With an Unfavorable Outcome
Source: Front Neurol. 2021 Jan 13;11:610192. doi: 10.3389/fneur.2020.610192 (PMC7839281; doi:10.3389/fneur.2020.610192)

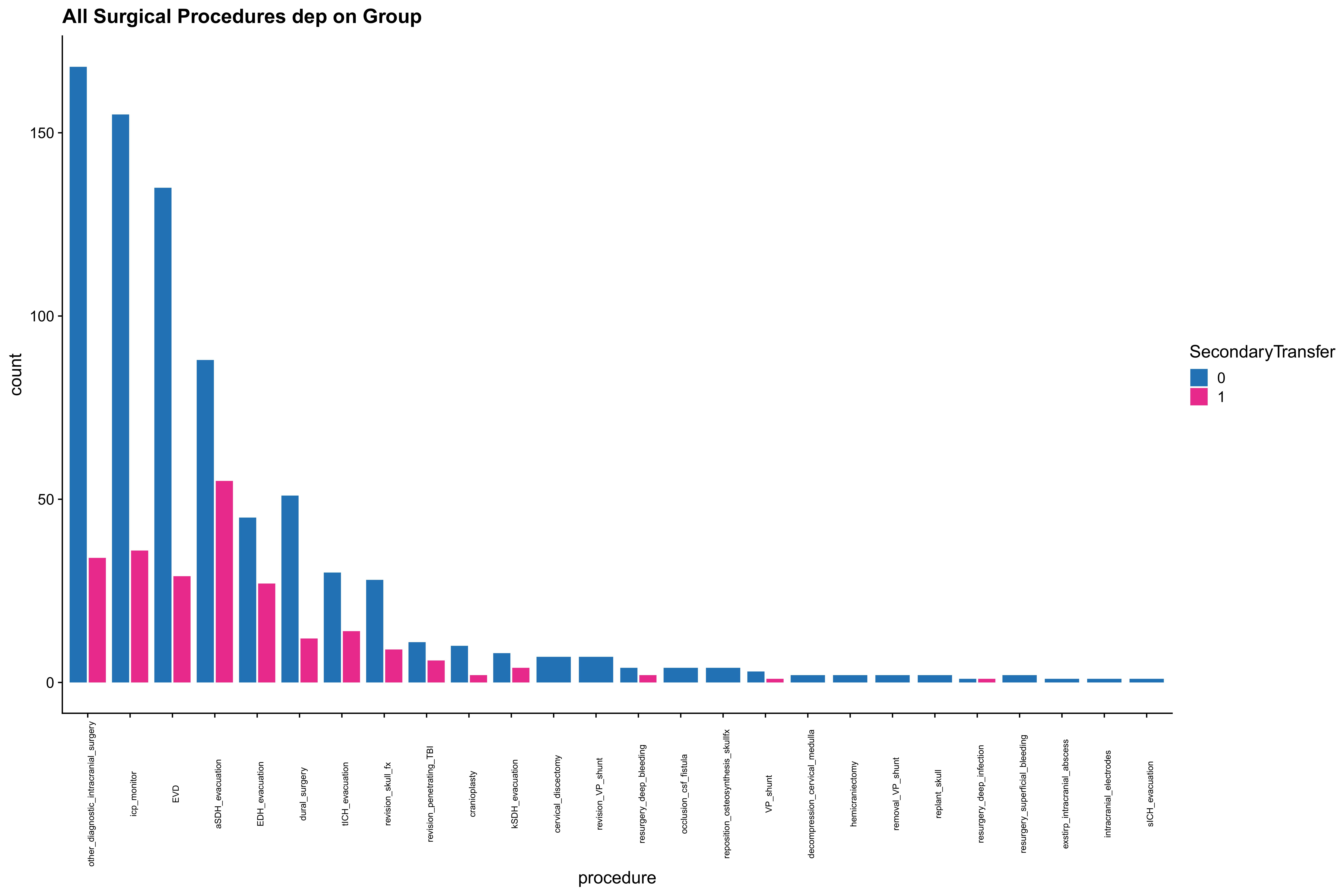

Supplement: Supplementary file 3 [file Image_1.TIF]

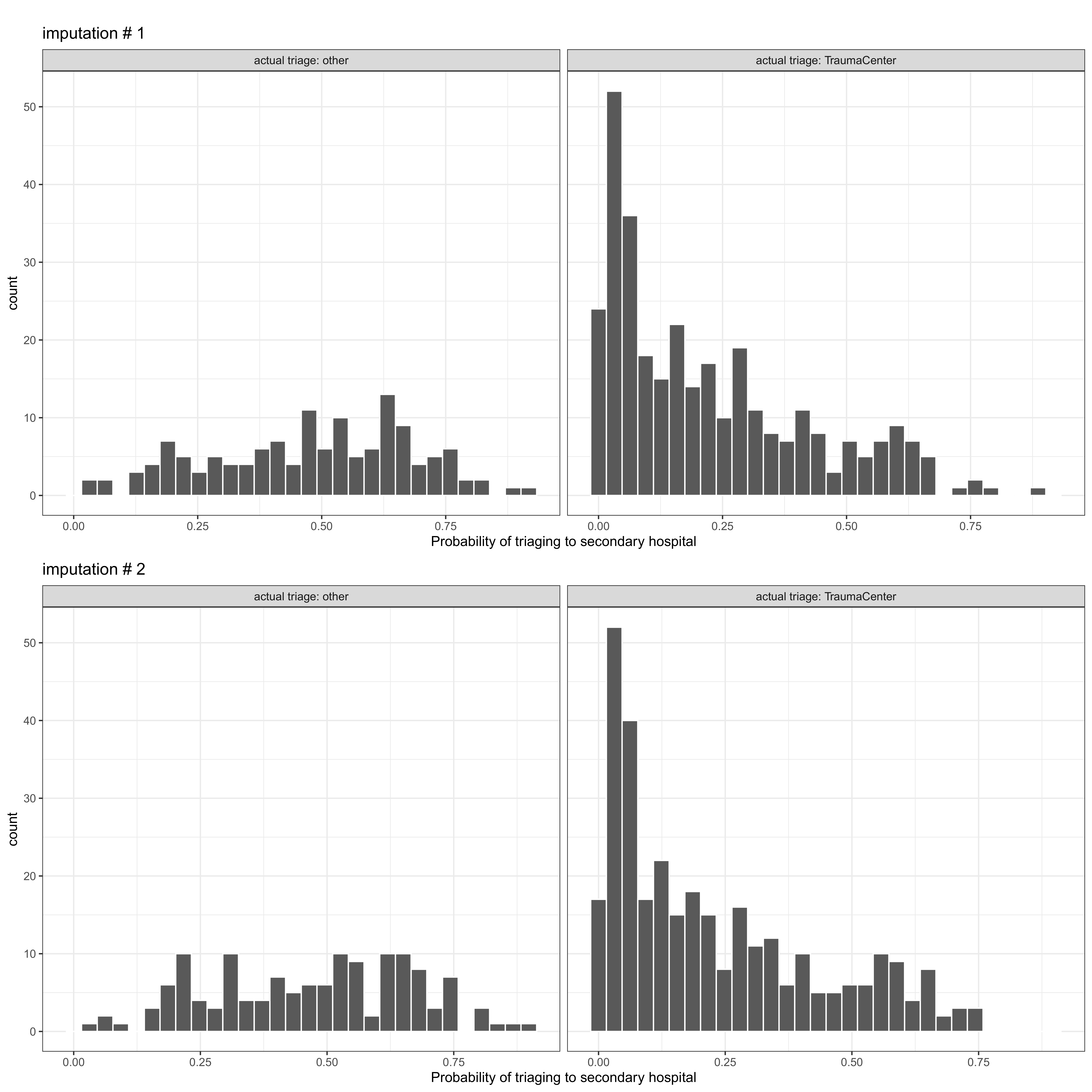

Supplement: Supplementary file 4 [file Image_2.TIF]

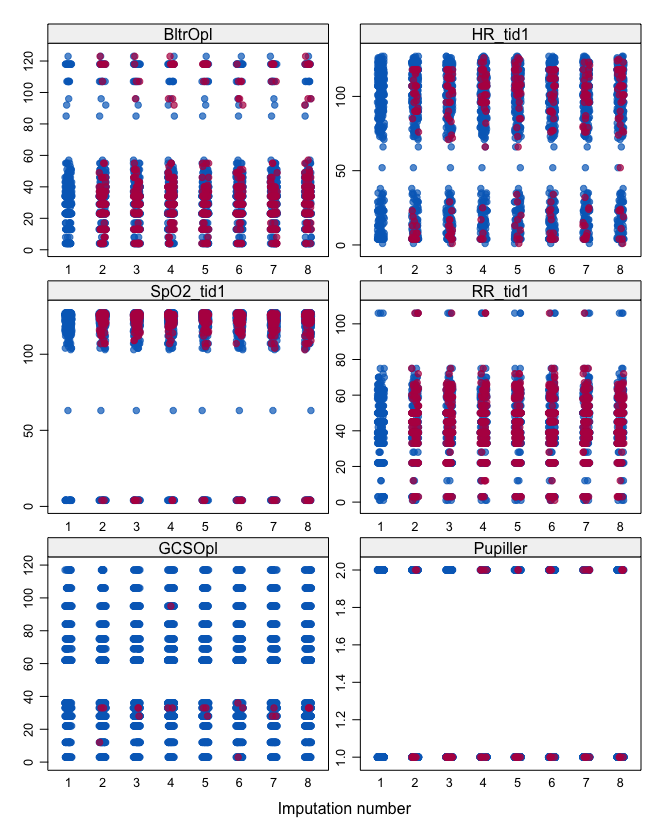

Supplement: Supplementary file 6 [file Image_4.TIF]
