# Supplementary material for: Delayed Neurosurgical Intervention in Traumatic Brain Injury Patients Referred From Primary Hospitals Is Not Associated With an Unfavorable Outcome
Source: Front Neurol. 2021 Jan 13;11:610192. doi: 10.3389/fneur.2020.610192 (PMC7839281; doi:10.3389/fneur.2020.610192)
Supplement: Supplementary file 7 [file Image_5.PDF]

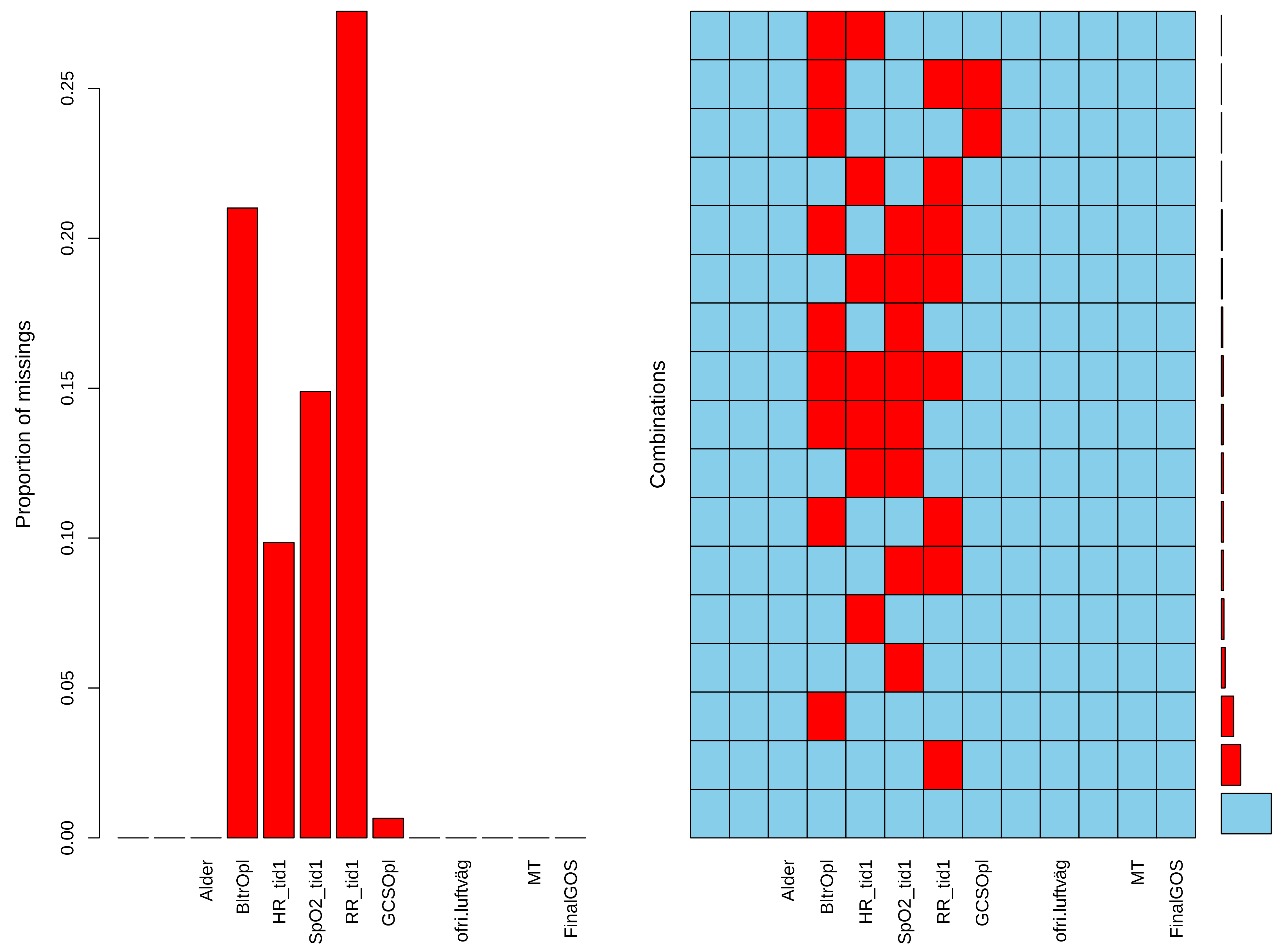

Proportion of missings
0.00
0.05
0.10
0.15
0.20
0.25
Alder
BltrOpl
HR_tid1
SpO2_tid1
RR_tid1
GCSOpl
ofri.luftväg
MT
FinalGOS
Combinations
Alder
BltrOpl
HR_tid1
SpO2_tid1
RR_tid1
GCSOpl
ofri.luftväg
MT
FinalGOS
