# Supplementary material for: Delayed Neurosurgical Intervention in Traumatic Brain Injury Patients Referred From Primary Hospitals Is Not Associated With an Unfavorable Outcome
Source: Front Neurol. 2021 Jan 13;11:610192. doi: 10.3389/fneur.2020.610192 (PMC7839281; doi:10.3389/fneur.2020.610192)
Supplement: Supplementary file 8 [file Table_1.docx]

**Supplementary Figure 1**

All surgical codes on the initial surgery that was performed, divided by transportation mode (y-axis; number of surgical codes registered). Sorted by frequency;

**“Other_diagnostic_intracranial_surgery”** (AAA99), commonly insertion of microdialysis or licox catheters.

**“icp_monitor”** (AAA20), insertion of intraparenchymal device to measure intracranial pressure (ICP).

**“EVD”** (AAF00), insertion of extraventricular drain (EVD).

**“aSDH_evacuation”** (AAD05), evacuation of an acute subdural hematoma.

**“EDH_evacuation”** (AAD00), evacuation of an acute epidural hematoma.

**“dural_surgery”** (AAK99), dural surgery.

**“tICH_evacuation”** (AAD15), evacuation of traumatic contusions.

**“revision_skull_fx”** (AAD40), revision of skull fracture.

**”revision_penetrating_TBI”** (AAD30), surgical revision of penetrating brain injury.

**“cranioplasty”** (AAK00), cranioplasty

**“kSDH_evacuation”** (AAD10), evacuation of chronic subdural hematoma

**“cervical_discectomy”** (ABC10), micro-surgical cervical discectomy

**“revision_VP_shunt”** (AAF20), revision of ventriculo-peritoneal shunt

**“resurgery_deep_bleeding”** (AWE00), secondary surgery due to deep cerebral bleeding

**“occlusion_csf_fistula”** (AAK40), surgical occlusion of cerebrospinal fluid fistula.

**“reposition_osteosynthesis_skullfix”** (AAK50), reposition and ostheosyntesis of skull fracture

**“VP_shunt”** (AAF05), insertion of a ventriculo-peritoneal shunt

**“decompression_cervical_medulla”** (ABC50 or ABC60), decompression of the cervical spinal cord

**“hemicraniectomy”** (AAK80), decompressive hemicraniectomy performed

**“removal_VP_shunt”** (AAF25), removal of ventriculoperitoneal shunt.

**“replant_skull”** (AAK85), reinsertion of previously removed bone flap.

**“resurgery_deep_infection”** (AWC00), secondary surgery due to deep cerebral infection.

**“resurgery_superficial_bleeding”** (AWD00), secondary surgery due to superficial cerebral bleeding.

**“exstirp_intracranial_abscess”** (AAM10), removal of intracranial abscess.

**“intracranial_electrodes”** (AAA35), insertion of subdural electrodes.

**“sICH_evacuation”** (AAB30), removal of spontaneous intracerebral hematoma.

**Supplementary Figure 2 – Imputation propensity index sample for being triaged to the trauma center or to a secondary hospital.**

Illustrating two data set imputations of probability to be triaged to either a secondary (left panels) or directly to the trauma center (right panels). A low number (x-axis) indicates a lower probability for transportation to a secondary hospital.

**Supplementary Figure 3 – Propensity score quality plots following data imputations**

Propensity score quality plots following data imputations reveals adequate data distribution.

**Supplementary Figure 4 – Multiple imputation by chained equations (mice) overview**

Figures depicting the existing data sets (blue) with the imputated (red) per imputation numbers (x-axis) for a number of different parameters. This illustrates a sufficient overlap indicating that the imputations rendered adequate data sets for comparisons. “BltrOpl” – Blood pressure at scene of accident. “HR_tid1” – Heart rate at the scene of accident. “SpO2_tid1” – oxygen saturation at scene of accident. “RR_tid1” – Respiratory rate at the scene of accident. “GCSOpl” – GCS at the scene of accident. “Pupiller” – Pupil reactivity.

**Supplementary Figure 5 – Missing data visualized**

Figures depicting the missing data, both in proportions and in the combinations used in the propensity analyses.

“BltrOpl” – Blood pressure at scene of accident. “HR_tid1” – Heart rate at the scene of accident. “SpO2_tid1” – oxygen saturation at scene of accident. “RR_tid1” – Respiratory rate at the scene of accident. “GCSOpl” – GCS at the scene of accident. “Pupiller” – Pupil reactivity, “Alder” = Age, “MT” = multitrauma. “Final GOS” = 6-12 months GOS.

**Supplementary Data 1 – Proportional odds analyses**

Data from the regressions performed in the manuscript.

**Supplementary Data 2 – Data missing from the propensity analyses**

The absolute and relative amount of missing data used in the propensity analyses.
